# Bettlägerigkeit und sozialräumliche Partizipation – eine Reflexion

**DOI:** 10.1007/s00391-025-02485-7

**Published:** 2025-08-27

**Authors:** Bianca Berger, Manfred Schnabel

**Affiliations:** 1 Hochschule Esslingen, Flandernstraße 101, 73732 Esslingen, Deutschland; 2 Ev. Hochschule Ludwigsburg, Paulusweg 6, 71638 Ludwigsburg, Deutschland

**Keywords:** Immobilität, Pflegeheim, Lebensraum Bett, Sozialraum, Raumsoziologie, Immobility, Nursing Home, Bed as living space, Social space, Sociology of space

## Abstract

**Hintergrund:**

Viele alte Menschen sind von Immobilität bis hin zur Bettlägerigkeit betroffen: Bettlägerigkeit stellt für die Betroffenen ein bedeutsames Thema dar, das erhebliche physische, psychische und soziale Risiken birgt und mit weitreichenden Folgen für die Partizipation der Personen einhergeht.

**Fragestellung:**

Welche Erkenntnisse können durch die Anwendung raumsoziologischer Konzepte bei der Beschreibung von Bettlägerigkeit und Partizipation in stationären Pflegeeinrichtungen gewonnen werden, und welche Wege zur Verbesserung von Partizipationschancen können sie aufzeigen?

**Methode:**

Grundannahmen der Raumsoziologie und das spezifische raumsoziologische Konzept von Martina Löw werden kurz vorgestellt. Das dort grundgelegte Verständnis von sozialen Räumen als Produkte einer Strukturen folgenden Praxis des Anordnens von Menschen und Dingen wird für die Analyse partizipationsfördernder und -hemmender Praktiken nutzbar gemacht. Dabei wird ein Verständnis von Partizipation, das Partizipationschancen als Verhältnis zwischen dem Vermögen zum Anordnen von Elementen zu sozialräumlichen Arrangements und dem Risiko des bloßen Angeordnetwerdens durch andere begreift, vorgestellt und grob skizziert. Eine Erprobung dieses Schemas erfolgt durch Anwendung auf zwei Fallbeispiele aus einer Beobachtungsstudie zur Gestaltung sozialer Interaktionen im Pflegeheim. Abschließend erfolgt eine erste Reflexion darüber, ob und welchen Beitrag die Raumsoziologie zur Partizipation von Bettlägerigen leisten kann.

**Ergebnisse:**

Die Anwendung einer raumsoziologischen Perspektive auf Fragen der Partizipation bei Bettlägerigkeit sensibilisiert für die Folgen des Platzierens von Menschen zueinander und zu den Dingen ihrer Umgebung. Durch die Anordnung von Bewohner*innen in Relation zu anderen Subjekten und Objekten wird ein Beziehungsraum mit spezifischen Wahrnehmungs- und Interaktionsmöglichkeiten geschaffen. Damit werden zugleich andere Wege, mit der Umwelt in Beziehung zu treten, versperrt. Um Partizipationschancen zu verbessern, müssen institutionalisierte Prozesse der Anordnung von Menschen und Dingen reflektiert und „Räume“ offen gestaltet werden.

**Diskussion:**

Es besteht Bedarf an Forschung zur Partizipation bettlägeriger Personen, die das Verhältnis von Raum, Partizipation und Immobilität auf der Ebene des Sozialraums fokussiert.

## Abstract (en)

**Background:**

Many older people are affected by immobility, which can eventually lead to becoming bedridden. Being bedridden is a significant issue for those affected as it carries considerable physical, psychological and social risks and has far-reaching consequences for a person’s ability to participate.

**Objective:**

Whether and to what extent can spatial sociology concepts be used and applied to demonstrate the effects on the participation of bedridden people in residential care facilities and what contribution can they make in this context?

**Method:**

The basic assumptions of the sociology of space and Martina Löw’s specific concept of spatial sociology are briefly introduced. The fundamental understanding of social spaces as products of a practice of arranging people and things according to structures is used to analyze practices that promote or hinder participation. In this context, a concept of participation is outlined that defines participatory opportunities as the relationship between the capacity to arrange elements into sociospatial configurations and the risk of being merely arranged by others. This analytical framework is then be tested by applying it to two case studies drawn from an observational study on the design of social interactions in nursing homes. Finally, an initial reflection is offered on whether and how spatial sociology can contribute to enhancing the participation of bedridden individuals.

**Results:**

Applying a spatial-sociological perspective to questions of participation in bedriddenness raises awareness of the consequences of placing people in relation to one another and to the objects in their surroundings. The arrangement of residents in relation to and among other “care-related objects” creates a relational space with specific possibilities for perception and interaction. Residents’ freedom of action within these functional arrangements is limited. In order to improve their opportunities for participation, institutional processes of arranging people and objects must be reflected upon and “environments” must be designed to be open.

**Discussion:**

There is a need for research on the participation of bedridden people that focuses on the relationship between space, participation and immobility at the spatiosociological level.

## Einführung

Rund 35 % der Personen im Alter von 70 Jahren und die Mehrheit der 85-Jährigen sind von Mobilitätseinschränkungen betroffen [4]. Bettlägerigkeit als ausgeprägteste Form ist mit sozialen und gesundheitlichen Risiken für die Betroffenen verbunden und ist deshalb von großer Bedeutung für die Pflege- und Gesundheitswissenschaften [1].

Ein Scoping-Review zum Thema Bettlägerigkeit von Berger et al. [2, S. 209] kommt zu dem Schluss, dass die bisherige Forschung den Fokus oft auf die „Risikofaktoren für die Entstehung von Immobilität und deren negativen Folgen“ legt. Andere Fragen, wie die Sichtweise der Betroffenen, die Gestaltung des Lebens im Bett oder der sozialen Partizipation bei ausgeprägter Immobilität, geraten dabei aus dem Blick. Will man Bettlägerigkeit als Teil der Lebenswirklichkeit einer wachsenden Zahl von Personen würdigen, ist eine Erweiterung der Forschungsperspektive notwendig.

Diese Arbeit behandelt Bettlägerigkeit deshalb unter dem Aspekt der Partizipation. Unter Verwendung raumsoziologischer Konzepte werden das Bett bzw. das Pflegezimmer als relationale Räume betrachtet. Partizipationschancen und -hemmnisse werden als Effekte der Aneignung von Räumen verstanden. Hierfür werden Fallbeispiele aus dem Forschungsprojekt PEBKO (Prävention in (teil-)stationären Pflegeeinrichtungen in den Handlungsfeldern Ernährung und Bewegung mittels partizipativer Konzeptentwicklung) zur Veranschaulichung herangezogen. Im Rahmen des Projekts PEBKO wurden teilnehmende und nichtteilnehmende Beobachtungen bei bettlägerigen Personen durchgeführt und Räumlichkeiten mittels Bildaufnahmen dokumentiert. Auszüge aus diesen empirischen Daten werden folgend für eine raumsoziologische Betrachtung genutzt.

Mit der Prämisse einer Wechselwirkung zwischen der Lebensrealität im Alter und dem Umfeld, in dem sich dieses Leben vollzieht, steht die Untersuchung in der Tradition ökogerontologischer Ansätze. Mit ihrer über die physische Wohnumwelt hinausreichenden Perspektive schließt sie an sozialräumliche Ansätze an. Die Fokussierung von Beziehungsnetzen zwischen Menschen *und* Dingen birgt wiederum Anschlüsse an praxistheoretische Zugänge zu sozialen Räumen [6].

Gegenüber der in sozial- und pflegewissenschaftlichen Kontexten häufig rezipierten leibphänomenologischen Perspektive auf Bettlägerigkeit stellt der gewählte Zugang eine Erweiterung dar [15, S. 21]. Zwar überwindet die Leibphänomenologie durch die Betonung der Dualität von physikalischem „Ortsraum“ und „Leibraum“ [15] eine simplifizierende Vorstellung von Bettlägerigkeit als fehlender Bewegungsfähigkeit in physikalischen Räumen; Raum wird vielmehr als das Äußere des Leibes und als Medium für die leibliche Erfahrung der Welt verstanden. Wenn Raum jedoch ausschließlich „vom subjektiven Gespür her“ [15] betrachtet wird, wird er als Arena für partizipative Aneignungs- und Mitbestimmungsprozesse ausgeblendet.

Zu kurz greifen u. E. auch Konzepte aus der Pflegepraxis, die zwar einerseits Mitbestimmung bei Bettlägerigkeit als Thema der Raumgestaltung aufgreifen, Raum dabei aber oft mit dem Pflegezimmer oder dem Pflegeheim gleichsetzen [10, 13]. Die Aspekte des Sozialraums als Beziehungsraum bleiben hier unberücksichtigt.

## Bettlägerigkeit und Immobilität

Der Begriff „Bettlägerigkeit“ wird uneinheitlich verwendet, bezieht sich aber meist auf Zustände der Gebundenheit bzw. der Fixierung von Menschen an Orte, z. B. an ein Bett oder ein Zimmer [11]. Zur Entstehung sind im deutschsprachigen Raum nach wie vor die Arbeiten Zegelins einflussreich [17, 18, 20]. Bettlägerigkeit entwickelt sich demnach prozesshaft als allmähliche Ortsfixierung. Anfangs zeigt sich eine zunehmende Verwiesenheit auf einen Ort, wie die Wohnung, ein Sitzmöbel oder einen Rollstuhl. In der letzten Phase des Prozesses kommt es dann zum dauerhaften Liegen im Bett [19]. Eine Reduktion der Selbstbestimmtheit und der Verlust der Selbstversorgungsmöglichkeiten gehen damit einher. Die Personen passen sich mit ihren Ansprüchen an, sie möchten nicht zur Last fallen. Ihre Teilhabe an der Gemeinschaft nimmt ab. Eine zunehmende Deprivation bestimmt ihren Alltag [1], und weitreichende körperliche Abbauprozesse können sich einstellen [5].

Immobilität ist eine der Ursachen von Bettlägerigkeit. Sie lässt sich, eng gefasst, als Einschränkung der physischen Funktionalität, weit gefasst, als Verlust freier und zielgerichteter Bewegungen, verbunden mit einer verlorenen Kontrolle über die Umwelt, beschreiben [11]. Dieser Kontrollverlust in Verbindung mit der Fixierung an einen Ort begründet die besondere Vulnerabilität bettlägeriger Personen in Bezug auf Partizipationschancen.

Die Prävalenz von Bettlägerigkeit in Pflegeheimen kann trotz der begrifflichen Unschärfe als hoch gelten. Nach einer repräsentativen österreichischen Studie liegt sie bei 49,8 % [12]. Für Deutschland sind ähnliche Verhältnisse anzunehmen.

## Sozialraum

Der Begriff Sozialraum beschreibt einen relationalen, von einer wechselseitigen Bedingtheit von manifester räumlicher Struktur und ihrer subjektiven Deutung und Nutzung konstituierten „Raum“ [12]. Dazu gehört einerseits die physische Wohnumgebung, in der sich das Leben eines Menschen hauptsächlich abspielt. Zum „Sozialraum“ wird ein physischer Ort allerdings erst durch die subjektive Bewertung und alltagspraktische Nutzung der vorhandenen personellen, materiellen und immateriellen Infrastrukturen durch die ihn „bewohnenden“ Menschen [7]. Sozialräume sind somit Beziehungsräume. Sie existieren als Netzwerk aus Verbindungen zwischen einer Person und einer Auswahl an subjektiv bedeutsamen Elementen in ihrem Umfeld.

Für die Realisierung von Partizipation spielt der Sozialraum als Sphäre unmittelbar erlebter Mitbestimmung und Teilhabe eine zentrale Rolle [12]. Dabei sind Menschen mit alters- oder krankheitsbedingten Mobilitätseinschränkungen bezüglich ihrer durch den Sozialraum vermittelten Partizipationschancen in vielfältiger Weise benachteiligt. Zum einen lassen Bewegungseinschränkungen den physischen Raum kleiner werden. Wenn das Leben im Pflegeheim stattfindet, gehen zudem über Jahrzehnte gestaltete und gepflegte sozialräumliche Arrangements einschließlich der darin enthaltenen materiellen und immateriellen Gewissheiten verloren.

## Sozialräume zwischen Handlung und Struktur

Löw, deren raumsoziologische Studien dieser Arbeit wichtige Impulse liefern, betont die Dualität von Struktur und Praxis bei der Konstitution von Räumen. Sie prägt dafür den Begriff der „(An)Ordnung“ [9]. Dieser verweist auf die Gleichzeitigkeit von verknüpften Elementen (Struktur) und der Verknüpfung der Elemente (Praxis) bei der Konstitution relationaler Räume. Gemeint ist, dass sich die Praxis des (An)Ordnens stets auf der Grundlage einer durch vorangegangene Handlungen entstandenen Ordnung vollzieht, sich auf diese bezieht, sie reproduziert und ggf. verändert. Angeordnet werden Dinge und Subjekte gleichermaßen. Menschen ordnen an und sind zugleich Teil der Anordnungen anderer Menschen [9]. Im Unterschied zu bloßen „Dingen“ sind sie allerdings dazu in der Lage, sozialräumliche Arrangements zu deuten, zu verändern oder aus eigenem Antrieb heraus zu verlassen.

Räume als relationale (An)Ordnungen entstehen nach Löw durch zwei aufeinander bezogene Prozesse. Zunächst werden mittels einer als *Spacing* bezeichneten Praxis Menschen und Dinge platziert und symbolisch markiert. So wird ein beliebiges Zimmer zum Behandlungsraum, wenn sich darin Therapeut*innen, Kranke, Möbel und Geräte einer spezifischen Ordnung folgend verteilen und Hinweisschilder dem entstandenen Konglomerat einen Namen geben. Abgeschlossen ist die Konstitution von Sozialräumen, wenn die solcherart entstandenen Muster durch einen zweiten, von Löw als *Syntheseleistung* bezeichneten Prozess in der Wahrnehmung zu einem „Raum“ verschmelzen [9]. Das Behandlungszimmer wird nicht als Ensemble von Menschen und Dingen, sondern als Summe seiner Teile wahrgenommen und abgespeichert.

Menschen schaffen Räume weder täglich neu noch ausschließlich aus eigenem Antrieb heraus. Raumbildende Praktiken sind vielmehr Ausdruck gesellschaftlich vermittelter Routinen. Auf Dauer gestellte Sozialräume entstehen und erhalten sich, wenn raumformendes Handeln sich auf institutionalisierte und vom konkreten Ort unabhängige gesellschaftliche Strukturen stützen kann [8, 9]. Sozialräume sind sowohl das Produkt von Strukturen (z. B. Regeln zu ihrer Gestaltung und Ressourcen zu ihrer Erhaltung) wie deren permanenter Reproduktion durch handelnde Personen [9].

## Partizipation als Raumaneignung

Gemeinhin werden zwei Aspekte von Partizipation unterschieden. Der erste zielt auf die Mitgestaltung der individuellen und sozialen Lebensbedingungen durch die „Teilnahme“ an Aushandlungs- und Entscheidungsprozessen. Teilnahme realisiert sich einerseits durch die Beteiligung an formalisierten Mitbestimmungsprozessen wie Wahlen, v. a. aber als aktive Mitgestaltung von Lebensorten und Gemeinschaften. Im Kontext Pflegeheim umfasst dieser Aufruf zur Mitgestaltung ausdrücklich auch die Bewohner*innen [16]. Der zweite Aspekt umfasst die „Teilhabe“ an materiellen und ideellen Gütern einer Gesellschaft. Gemeint ist die anteilige Verfügungsgewalt über gesellschaftliche Ressourcen zur Verwirklichung eigener Lebensentwürfe und Entwicklungsprozesse. Beide Aspekte sind aufeinander bezogen. Die Teilnahme an der Schaffung sozialer Lebensbedingungen (in Bezug auf den Zugang zu Ressourcen oder die Absicherung von Risiken) schafft erst die Grundlage für die Verwirklichung von Teilhabe [14].

In einem sozialräumlichen Bezugsrahmen lassen sich Partizipationschancen über das Konzept der Raumaneignung fassen. Der Begriff der „Aneignung“ beschreibt das In-Beziehung-Treten eines Menschen zur Welt. Dies geschieht, einfach gesagt, durch einen wechselseitigen Vermittlungsprozess. Menschen werden Teil der Welt, indem sie sich deren Ordnung erschließen und in sich abbilden. Gleichzeitig wird die Welt dadurch zu einem Teil des Menschen, erfährt eine subjektive Deutung und Veränderung [7]. Im hier gewählten Kontext zielt Aneignung auf das Zueigenmachen von durch Spacing objektivierten, durch den Einsatz von Ressourcen stabilisierten und durch beständiges Handeln aufrechterhaltenen sozialräumlichen Konfigurationen. Dabei ist zu beachten, dass auch die Aneignung von bestehenden Sozialräumen ihrerseits raumkonstituierendes Handeln ist. Sie bringt über die physische oder symbolische Neuordnung bestehender Beziehungsmuster neue Konfigurationen von Menschen und Dingen und somit neue Sozialräume hervor. Die Aneignung von Sozialräumen hat somit zwei Aspekte: erstens die Auseinandersetzung der Subjekte mit bestehenden Objektivationen und ihre Bewertung als „aneignungswürdig“ und zweitens die damit einhergehende Produktion neuer Räume (die von anderen Subjekten erneut angeeignet werden müssen). Raumaneignung bedeutet folglich nicht, einen physischen Raum zu besetzen, sondern Räume handelnd zu erschaffen, indem bestehende Konfigurationen für die Realisierung eigener Handlungsentwürfe erschlossen werden. Probleme der Raumaneignung sind deshalb auch keine Probleme des Raums, sondern des raumkonstituierenden Handelns [6]. Aus dieser Warte können Fragen nach dem Willen zur Raumaneignung, nach den dafür erforderlichen materiellen und immateriellen Ressourcen und nach deren ggf. ungleichen Verteilung zwischen den Subjekten gestellt werden.

## Partizipation und Bettlägerigkeit

Die vorgestellten Konzepte sind gut geeignet, Partizipationschancen bettlägeriger Personen auf der Ebene des Sozialraums zu fassen. Den Sozialraum als Konglomerat aus Menschen und unbelebten Objekten zu betrachten, rückt die Platzierung von Menschen innerhalb räumlicher Gegebenheiten (Zimmer, Treppen, Gänge) in den Blick, insbesondere, wenn zugewiesene Positionen nicht aus eigenem Antrieb verlassen werden können. Rollstühle und Pflegebetten können vor diesem Hintergrund als Platzierungshilfen betrachtet werden, für Bewohner*innen und vielleicht mehr noch für das Personal. Partizipation wird zu einer Frage der Einflussnahme auf Positionierungen. Die Begriffe „Spacing“ und „Synthese“ akzentuieren die Rolle des Subjekts bei der Konstitution von Sozialräumen, sowohl bei deren Hervorbringung wie auch bei deren dauerhafter Abspeicherung als „Raum“. Pflegeheime sind so verstanden nicht reine Architektur, die hingenommen werden muss; vielmehr entstehen sie als relationales Setting von „Räumen“ durch die alltägliche und regelgeleitete Nutzung durch Personal und Bewohner*innen. Der Begriff der Anordnung führt zudem eine Unterscheidung zwischen Anordnenden und Angeordneten ein. Daran anschließend sensibilisiert das Konzept der Raumaneignung dafür, dass das Verhältnis zwischen diesen beiden Gruppen nicht immer ausgewogen ist. Raumkonstituierende Prozesse sind machtbesetzt und können soziale Ungleichheiten verstetigen.

Partizipation, verstanden als tätige Mitwirkung der Pflegeheimbewohner*innen an der Gestaltung der Institutionen, ist somit die Frage nach den Chancen zur Aneignung von Räumen angesichts verfestigter sozialräumlicher Beziehungssysteme zwischen den Personen, Gegenständen und Orten im Pflegeheim [14]. Dies erfordert neben dem Willen zur Aneignung (Teilnahme) auch die Verfügungsgewalt über materielle und immaterielle Ressourcen (Teilhabe). Werden Bewohner*innen dagegen in einer Art und Weise „verortet“, die ihnen die Aufrechterhaltung subjektiv bedeutsamer Beziehungen erschwert bzw. diese überschreibt, lässt sich dies als Fehlen von Partizipationschancen interpretieren. Im Zentrum steht somit die Frage nach dem Verhältnis zwischen Anordnenden und Angeordneten oder anders: zwischen machtbesetzten Prozessen der Raumaneignung und solchen der eher machtlosen Anpassung an die dadurch entstandenen neuen Beziehungssysteme. In diesem Sinn können nun Partizipationschancen (überwiegend) bettlägeriger Menschen anhand von zwei Fallbeispielen reflektiert werden.

## Anordnung und Raumaneignung – Fallbeispiel 1

Das „Pflegezimmer“ wird betreten. Das Bett mit der liegenden Person steht präsent im Raum. Persönliche Bilder und Hinweise, die bei der Pflege zu beachten sind, sind hinter dem Bett der bettlägerigen Person angebracht, sodass sie im Blickfeld der Pflege, aber nicht der Person sind. Oder sie stehen in einer Entfernung, die es dem Betroffenen nicht oder nur teilweise erlaubt, die Gesichter oder die Situationen auf den Fotografien zu erkennen. Eigene Möbel werden als Abstellfläche für Pflegeartikel genutzt. Ein offener Karton mit Pflegeutensilien steht zudem im Raum. Die Gestaltung des Lebensraumes wirkt funktional. Die Aufrichthilfe am Bett („Bettgalgen“) ist beispielsweise dauerhaft im Blick der pflegebedürftigen Person, auch wenn diese nicht genutzt wird. Zudem werden Pflegeutensilien auf dem Tisch (z. B. Desinfektionsmittel, Pflegehandschuhe, Dokumentation) platziert. Auf einem Stuhl wird eine Inkontinenzeinlage gelagert. Auf einem anderen Stuhl Decken und Lagerungsutensilien. Der Fernseher ist angeschaltet und läuft über lange Zeitspannen.

### Reflexion

Gemäß den genannten Prämissen kann das beschriebene Ensemble aus pflegebedürftiger Person, Personal, Raum und Gegenständen als relationaler, durch Spacing und Synthese gebildeter Sozialraum aufgefasst werden. Er entsteht, wenn durch das spezifische Anordnen von Pflegebett, Stau- und Lagerungsraum für Pflegeartikel, hauseigenen und privaten Möbeln, Bewohner*in und Personal ein Ensemble etabliert und ggf. durch das Anbringen eines Namensschildes an der Tür symbolisch markiert wird. Wahrgenommen wird dieser Raum – zumindest beim Lesen der Beschreibung – nicht als das Zimmer von Person X, sondern als typisches Ensemble aus Dingen und Menschen, das mit ähnlichen Räumen in eine Reihe gestellt und von anderen Bereichen des Pflegeheims (Speisesaal, Pflegebad, Kapelle) unterschieden werden kann. Die Platzierung der pflegebedürftigen Person innerhalb eines Ensembles von anderen „Objekten“ durch das Pflegepersonal fördert mutmaßlich ihre Objektivierung. Dafür spricht die funktionale Gestaltung ihres Lebensraumes oder der Fernseher im Dauerbetrieb, der anscheinend unbeeinflusst vom Willen der pflegebedürftigen Person zur bloßen optisch-akustischen Kulisse der Pflegehandlungen wird.

Die beschriebene Situation lässt sich unschwer als eine Form der Raumaneignung beschreiben. Das Pflegepersonal übernimmt den eigentlich der pflegebedürftigen Person vorbehaltenen Raum, indem es Beziehungssysteme zwischen sich, ihr und den aus Pflegesicht relevanten Gegenständen herstellt und damit einen Raum zur Umsetzung eigener Handlungspräferenzen etabliert. Etwaige Beziehungssysteme der Person, z. B. zu den eigenen Möbeln oder Bildern, werden überschrieben. Die solcherart „enteignete“ Person wird Teil des Handlungsraumes anderer. Dass sie in Entscheidungsprozesse einbezogen wird, ist nicht erkennbar. Der Raum ist funktionalen Zwecken angepasst, als privater Lebensraum verblasst er mangels subjektiver Merkmale zur Unkenntlichkeit.

## Anordnung und Weltbezug – Fallbeispiel 2

Eine bettlägerige Person wird vom Bett in einen Liegewagen transferiert und an einem Fenster in der Gemeinschaftsküche platziert. Dem Ortswechsel geht keine Absprache voraus; als Teil der täglichen Routinen wird er sprachlos abgewickelt. Vom neuen Platz aus kann die Person auf die Straße sehen, sieht aber die Tischgemeinschaft nicht und kann mit den Personen nicht sprechen, ist abgewendet vom Alltagsleben. Die Mitbewohner*innen nehmen die abseits liegende, bettlägerige Person zwar zur Kenntnis, diese wird jedoch nicht in den Alltag und die Aktivitäten integriert.

### Reflexion

Auch hier wird die Konstitution eines sozialen Raumes beschrieben. Die Platzierung eines Menschen durch das Personal verändert ein bestehendes Ensemble aus Menschen und Dingen und schafft ein neues, das andere Zwecke befördert. Wichtig ist, dass hier nicht nur ein Liegewagen mit dem pflegebedürftigen Menschen verschoben wird, sondern ein ganzes Set an möglichen Weltbezügen. In Beziehung treten und Aneignen des sozialen Umfeldes sind für die verschobene Person nur in dem Rahmen möglich, den die Platzierung zulässt – als Blick durchs Fenster. Sozialkontakte können nicht unabhängig vom anordnenden Personal hergestellt werden. Die Person wird angeordnet, sie findet ihren Platz im Gefüge eines routinierten Pflegeprozesses.

## Partizipative Aneignungsprozesse verhindern oder verbessern

Zur Aufrechterhaltung von Teilnahme und Teilhabe bei Bettlägerigkeit wird in der Literatur eine Reihe von Vorschlägen unterbreitet: So kann Handlungsfähigkeit erhalten werden, indem das Bett bzw. das Pflegezimmer zur persönlichen Schaltzentrale, in der alle wichtigen „Hebel“ zur Alltagsgestaltung (Telefon, Fernbedienungen etc.) in Reichweite sind, wird [15]. Etablierte soziale Beziehungsmuster können durch die Erleichterung von Außenkontakten auch vom Bett aus aufrechterhalten werden, z. B. durch digitale Kommunikationsformen [15]. Machtaspekte bei der Platzierung in Räumen bleiben hier allerdings unberücksichtigt. Nach der hier gewählten raumsoziologischen Fundierung sind auf Dauer gestellte relationale Räume, inklusive ihrer Reproduktion, durch Handlungen in gesellschaftliche Strukturen, die ihnen vorausgehen, eingebettet. Schon das Errichten spezieller Häuser für die Pflege alter Menschen, das Erlassen von Vorschriften zu ihrer Form und Funktion oder die Einführung von Standards und Leitlinien für ihren Betrieb lassen sich als Spacing und Synthese auffassen [15]. Dabei werden Machtverhältnisse reproduziert [9].

Um Raumaneignung zu ermöglichen, müssen Gestaltungsmöglichkeiten deshalb die physikalischen Räume, inklusive der damit verbundenen sozialräumlichen Ordnungen, selbst umfassen. Nach Bleck und Höppner könnten z. B. Räume geschaffen werden, die von Bewohner*innen selbstorganisiert und frei von Vorgaben genutzt werden können. Räume mit offenen Strukturen könnten Partizipationsmöglichkeiten verbessern, indem sie ausreichend Wahlmöglichkeiten in Bezug auf Sitzgelegenheiten, Beschäftigung oder weiterführende Wege eröffnen [3]. Bezogen auf bettlägerige Menschen kommen diese Vorschläge allerdings ebenfalls an Grenzen.

Bleibt die Frage, welchen Beitrag die hier vorgestellten Konzepte für die Einlösung des Anspruchs auf Partizipation auch bei Bettlägerigkeit leisten können. Zu nennen ist hier v. a. das kritische Potenzial des Ansatzes. Von seiner Warte aus können z. B. Vorschläge aus der Praxis, Partizipation durch sorgfältig zusammengestellte Tischgemeinschaften [16] von Bewohner*innen zu fördern, unschwer als eine Form der Anordnung identifiziert werden. Partizipativ wäre das nur, wenn es der Verwirklichung persönlicher Prämissen der solcherart Platzierten dient, von Ihnen bewusst eingefordert und v. a. eigenen Vorstellungen entsprechend verändert werden kann. Um den Anspruch auf Partizipation wirklich einzulösen, müssen dagegen die hier deutlich werdenden Asymmetrien zwischen Anordnenden und Angeordneten, soweit möglich, ausgeglichen werden. Ob und wie dies gelingen kann, ist eine Frage für zukünftige Untersuchungen.

## Ausblick

Die raumsoziologische Perspektive erweist sich grundlegend als fruchtbar bei der Betrachtung von Partizipation und Bettlägerigkeit im stationären Kontext. Die Integration von Artefakten wie Liegemöbeln, Tischen, Bettgalgen etc. und der Gegebenheiten des physischen Raums in die Analyse ermöglicht es, die Öffnung oder Schließung von Weltbezügen und damit die Möglichkeit zur Teilnahme durch Aneignung subjektiv bedeutsamer sozialer Räume allein als Effekte der Platzierung im Raum und der Verfügungsgewalt über Mobilitätshilfen zu betrachten. Gerade bei der Betrachtung von Partizipationschancen immobiler, stärker auf den physischen Raum verwiesener Menschen ist dies von Bedeutung. Die Konzepte Spacing und Syntheseleistung können beschreiben, wie Muster entstehen und Wirklichkeit konstituieren, indem sie die Wahrnehmung und Erinnerung der Akteure prägen und sich dadurch zugleich gegen Veränderung immunisieren. In Kombination mit dem Begriff der Raumaneignung ergibt sich ein theoretisches Fundament zur Erfassung von Verdinglichungsprozessen in stationären Settings. Strukturen folgende Handlungsroutinen von Fachkräften und Bewohner*innen besetzen, erschaffen und tradieren Räume, in denen Menschen und Dinge zu Teilen eines Ensembles verschmelzen.

Mit den hier vorgestellten Konzepten ist das raumsoziologische Begriffsrepertoire lange nicht erschöpft. So verfügen institutionalisierte Beziehungsräume über „Atmosphären“, verstanden als kulturell vermittelte und affektiv aufgeladene Wahrnehmungsfilter für sozialräumliche Arrangements [9]. Typische Settings eines Pflegeheimes erzeugen über die ihnen anhaftende Atmosphäre Gefühle, z. B. Resignation oder Hoffnungslosigkeit beim Blick auf ihre Bewohner*innen. Auf der Ebene sinnlicher Wahrnehmung tragen sie zum Einverständnis von Außenstehenden, Personal und ggf. der Bewohner*innen selbst mit deren machtlosen Stellung bei und fördern die Verfestigung bestehender Ungleichverhältnisse [6, 9].

Der Forschungsbedarf im Bereich Partizipation bettlägeriger Personen bleibt weiterhin evident. Eine umfassendere Perspektive, die sozialräumliche Aspekte von Bettlägerigkeit berücksichtigt, kann helfen, alltägliche Praktiken kritisch zu reflektieren und andere Lösungen für mehr Teilnahme und Teilhabe zu entwickeln. Zukünftige Studien sollten untersuchen, wie diese nicht nur auf der Ebene der physischen, sondern auch der in praktischen Vollzügen erzeugten sozialen Raumgestaltung verbessert werden kann.

## Fazit für die Praxis

- Raumsoziologische Überlegungen sensibilisieren für alltägliche Aneignungsprozesse von Pflegenden im Rahmen routinierter Versorgungsprozesse.
- Eine Verbesserung von Partizipation lässt sich nicht einseitig durch Maßnahmen des Pflegepersonals erreichen.
- Entscheidungsoptionen von bettlägerigen Personen und Gestaltungsspielräume im Lebensraum Bett werden im Gegenteil in dem Maße eröffnet, indem institutionalisierte Handlungsabläufe relativiert bzw. aufgegeben werden.

## Data Availability

Der Artikel enthält keine Daten, die zur Verfügung gestellt werden.
